# *Pneumocystis jirovecii* pneumonia (PCP) PCR-negative conversion predicts prognosis of HIV-negative patients with PCP and acute respiratory failure

**DOI:** 10.1371/journal.pone.0206231

**Published:** 2018-10-25

**Authors:** Ji Soo Choi, Sang Hoon Lee, Ah Young Leem, Joo Han Song, Song Yee Kim, Kyung Soo Chung, Ji Ye Jung, Young Ae Kang, Young Sam Kim, Joon Chang, Moo Suk Park

**Affiliations:** Division of Pulmonology, Department of Internal Medicine, Severance Hospital, Yonsei University College of Medicine, Seoul, Republic of Korea; Central University of Tamil Nadu, INDIA

## Abstract

**Background:**

*Pneumocystis jirovecii* pneumonia (PCP) is often fatal in human immunodeficiency (HIV)-negative patients and typically presents with respiratory insufficiency. Predicting treatment failure is challenging. This study aimed to identify prognostic factors and examine PCP polymerase chain reaction (PCR)-negative conversion in non-HIV PCP patients with respiratory failure.

**Method:**

We retrospectively enrolled 81 non-HIV patients diagnosed with and treated for PCP with respiratory failure in the intensive care unit at a tertiary hospital over a 3-year period. PCP was diagnosed via nested PCR-mediated detection of *Pneumocystis jirovecii* in induced sputum samples, endotracheal aspirates, and bronchoalveolar lavage fluids. PCP PCR was performed weekly to check for negative conversion.

**Results:**

The overall survival rate was 35.8%. Seventy-four patients (91.3%) required mechanical ventilation, and 6 (7.4%) required high-flow nasal oxygen treatment. The PCP PCR-negative conversion rate was 70.5% (survivors, 97%; non-survivors, 63.5%); the median time to conversion was 10 (7.0–14.0) days. On univariate analysis, the APACHE II score (*p* < 0.001), renal failure requiring renal replacement therapy (*p* = 0.04), PCP PCR-negative conversion (*p* = 0.003), and the PaO_2_/FiO_2_ ratio (first 24 hours) (*p* < 0.001) significantly correlated with mortality. On multivariate analysis, PCP PCR-negative conversion (hazard ratio, 0.433; 95% confidence interval, 0.203–0.928; *p* = 0.031) and the PaO_2_/FiO_2_ ratio (first 24 hours) (hazard ratio, 0.988; 95% confidence interval, 0.983–0.993; *p* < 0.001) independently predicted prognosis.

**Conclusions:**

Determination of PCP PCR-negative conversion and PaO_2_/FiO_2_ ratios may help physicians predict treatment failure and mortality in non-HIV PCP patients with respiratory failure.

## Introduction

*Pneumocystis jirovecii* pneumonia (PCP) is the most prevalent opportunistic infection in patients with human immunodeficiency virus (HIV) positivity [1]. However, the incidence of PCP in HIV patients has recently declined due to anti-retroviral therapy and prophylaxis [2,3]. Conversely, incidence rates in non-HIV and immunocompromised patients have risen, as more of these patients receive chemotherapy or immunosuppressants [4,5]. PCP typically presents with abrupt respiratory insufficiency. It is more often fatal and progresses more rapidly in non-HIV patients than in HIV patients [1,6,7], and longer times from admission to diagnosis and treatment in the former correlate with reduced survival rates [8].

The treatment response in PCP patients is difficult to determine. Although usually based on laboratory and radiological findings and clinical presentations, the findings sometimes indicate a different response than the presentations [9], and the best evaluation method is unclear. Several studies have investigated incidence, prognosis, and risk factors in non-HIV PCP patients, with differing results. Given the disease’s severity, rapid progression, and poor prognosis [5,10–12], identifying valid prognostic indicators in these patients is needed. This study aimed to identify prognostic factors for PCP and to examine PCP polymerase chain reaction (PCR)-negative conversion in non-HIV PCP patients with respiratory failure.

## Materials and methods

### Patients

We retrospectively reviewed the medical records of patients admitted to the medical intensive care unit (ICU) at Severance Hospital, a tertiary care university hospital in South Korea, from January 1, 2013 to December 31, 2015. Institutional review board approval for this study was provided by Severance Hospital (IRB 4-2016-0362), and informed consent was waived. Patient inclusion criteria were as follows: ICU-admitted non-HIV patients with PCP and respiratory failure requiring a ventilator or high-flow nasal oxygen treatment were included in this study. Patients who were positive for PCP PCR but not treated for PCP pneumonia due to a low clinical probability of true PCP infection were excluded from this study. Those patients did not present typical pneumonia symptoms and their imaging studies did not present typical PCP pneumonia findings. Additionally, we excluded patients with combined invasive aspergillosis, pulmonary hemorrhage, interstitial pneumonia with viral or fungal infection, and acute exacerbation of interstitial lung disease. And, there are no patients diagnosed and treated to pulmonary tuberculosis.

### Diagnosis of PCP

PCP was diagnosed by 3 criteria. First, the clinical symptoms of pneumonia (fever, cough, sputum, and dyspnea) were apparent, and the patients were immunocompromised from chemotherapy, use of immunosuppressants, or long-term steroid use. Second, ground glass opacities, reticular opacities, or septal thickening was observed on chest computed tomography (CT) scans. Third, PCP PCR assays of sputum samples, endotracheal aspirates, or bronchoalveolar lavage (BAL) fluids demonstrated the presence of *Pneumocystis jirovecii* DNA. After PCP PCR, the patients were followed up weekly to check for negative conversion; these tests were taken via endotracheal aspirates in patients with intubation and sputum samples in patients without intubation.

### PCP PCR

We performed the nested PCP PCR using a Thermal Cycler S1000 (Bio-Rad, Hercules, CA, USA) using the following primers: The first step was pAZ102-E (5'-GAT GGC TGT TTC CAA GCC CA-3') and pAZ102-H (5'-GTG TAC GTT GCA AAG TAC TC-3'), and the second step was pAZ102-X (5'-GTG AAA TAC AAA TCG GAC TAG G-3') and pAZ102-Z (5'-TCA CTT AAT ATT AAT TGG GGA GC-3'). The first PCR step was conducted in a total 20.0 μL volume including 1.0 μL of 20 pM primer pAZ102-E, 1.0 μL of 20 pM primer pAZ102-H, 1.0 μL of foramide, 15 μL of H₂O PCR grade (distilled water), and 2.0 μL of DNA template. The PCR amplifications were conducted as follows: 2 minutes at 94°C, then 35 cycles of 94°C for 30 seconds, 55°C for 30 seconds, 72°C for 20 seconds, and a final step at 72°C for 5 minutes. The second PCR step was performed in a total 20.0 μL volume including 1.0 μL of 20pM primer pAZ102-X, 1.0 μL of 20 pM primer pAZ102-Z, 1.0 μL of foramide, 15 μL of H_2_O PCR grade (distilled water), and 2.0 μL of DNA template. The PCR amplifications of the second step were conducted as follows: 2 minutes at 94°C, then 35 cycles of 94°C for 30 seconds, 53°C for 30 seconds, 72°C for 20 seconds, and a final step at 72°C for 5 minutes. The detections of PCR products were checked with 2.0% agarose gel, and a positive result was reported when a 260-bp sequence was detected.

### Statistical analysis

All statistical analyses were performed using IBM SPSS version 23.0 software (IBM Corp., Armonk, NY, USA). Continuous variables were presented as mean and standard deviation for normal distributions and as median and interquartile ranges for non-normal distributions. Categorical variables were expressed as frequency and percentages. Continuous variables were compared between survivors and non-survivors using the Student’s *t*-test and Mann-Whitney U test, and categorical variables were compared using the chi-squared test. A multivariate Cox proportional hazards model was used to analyze prognostic factors. Overall survival rates were estimated via Kaplan-Meier analysis. *p* < 0.05 was considered statistically significant.

## Results

**Fig 1:**
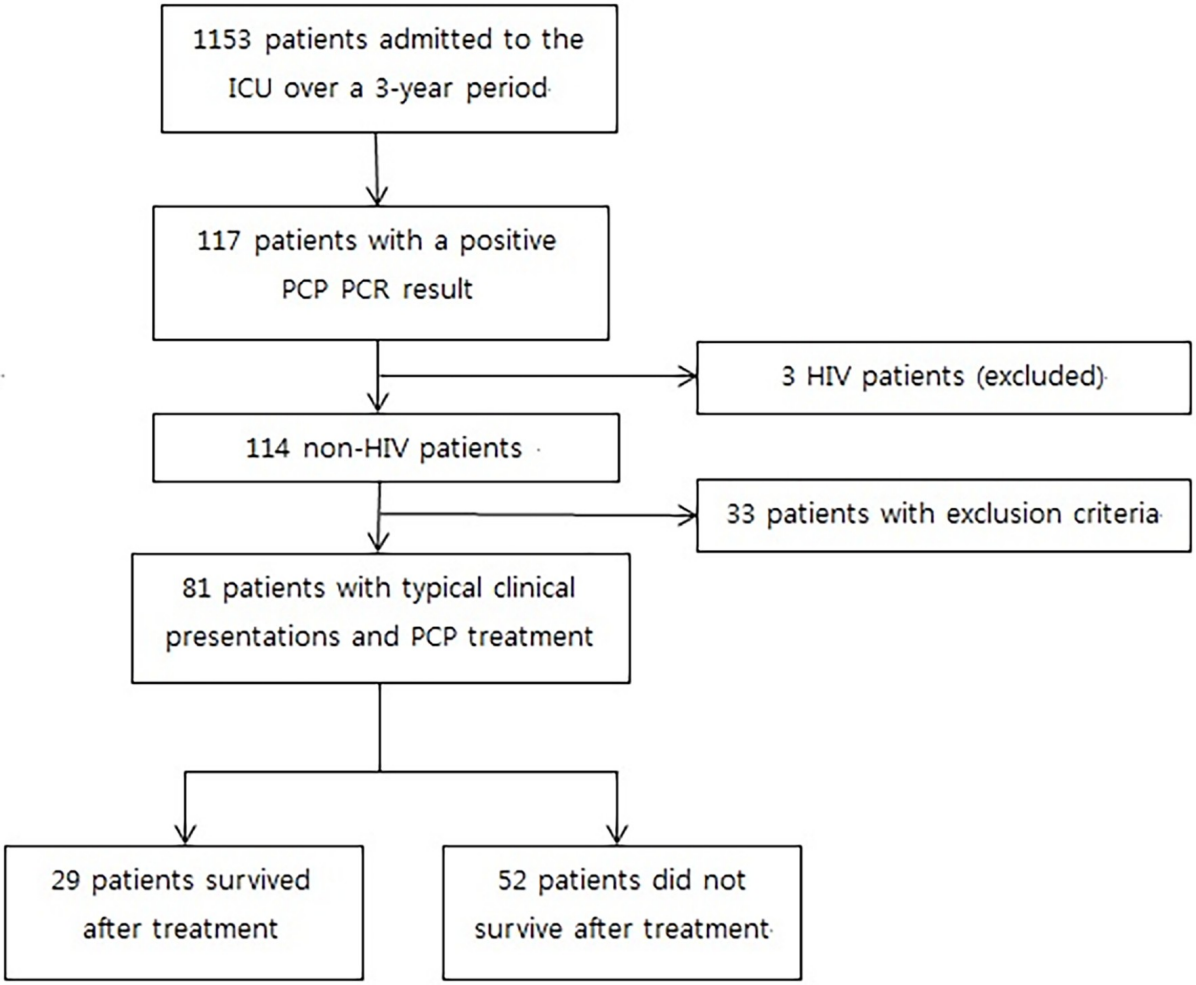
Flowchart showing patient selection. ICU, intensive care unit; PCP, *Pneumocystis jirovecii* pneumonia; PCR, polymerase chain reaction; HIV, human immunodeficiency virus.

Among the 1153 patients admitted to our ICU, 117 had a positive PCP PCR result in their sputum samples or BAL fluids (Fig 1). Three PCP patients who were diagnosed with HIV infection after PCP diagnosis were excluded, and 33 patients were excluded for meeting other exclusion criteria. The remaining 81 patients with PCP that were HIV-negative and had acute respiratory failure and atypical pneumonia were treated for PCP and were the subjects of our study.

### Baseline characteristics

The study cohort consisted of 51 men (63.0%) and 30 women (37.0%), with a median age of 60.9 (50.0–71.5) years (Table 1). Thirty-six patients (44.4%) were current or former smokers. The median APACHE II score was 21.1 (16.0–25.0). The most common comorbidity was hypertension (n = 46, 56.8%), followed by chronic renal failure (n = 25, 30.9%). The most common underlying disease was a solid malignancy (n = 29, 35.8%), followed by a status of solid organ transplantation (n = 16, 19.8%) and a hematologic malignancy (n = 14, 17.3%). Among the 7 patients with a rheumatologic disease, 5 had vasculitis and 2 had rheumatoid arthritis from long-term use of steroids or immunosuppressants. Twenty-seven patients (33.3%) had used corticosteroids for >3 months, 40 (53.1%) had undergone chemotherapy or radiotherapy, and 20 (24.7%) had received immunosuppressants.

**Table 1:** Baseline characteristics of non-HIV PCP patients with respiratory failure.

Variable	n = 81
**Median age, years (IQR)**	60.9 (50.0–71.5)
**Male sex, n (%)**	51 (63.0%)
**Ever smoking, n (%)**	36 (44.4%)
**APACHE II score, median (IQR)**	21.1 (16.0–25.0)
**Comorbidity, n (%)**	
**Hypertension**	46 (56.8%)
**Diabetes mellitus**	23 (28.4%)
**Congestive heart failure**	6 (7.4%)
**Cerebrovascular disease**	4 (4.9%)
**Chronic renal failure**	25 (30.9%)
**Chronic liver disease**	8 (9.9%)
**Chronic obstructive lung disease**	4 (4.9%)
**Underlying disease, n (%)**	
**Solid malignancy**	29 (35.8%)
**Hematologic malignancy**	14 (17.3%)
**Solid organ transplantation**	16 (19.8%)
**Rheumatologic disease**	7 (8.6%)
**Others†**	15 (18.5%)
**Previous treatment, n (%)**	
**Chemotherapy or Radiotherapy**	43 (53.1%)
**Corticosteroid use >3 months**	27 (33.3%)
**Cyclosporine, mycophenolate, tacrolimus, sirolimus, or methotrexate**	20 (24.7%)
**CMV quantitative real-time PCR positive in blood sample, n (%)**	43 (53.1%)
**Second-line treatment, n (%)‡**	19 (23.5%)
**Respiratory samples, n (%)**	
**BAL fluids**	5 (6.2%)
**Endotracheal aspiration**	51 (63.0%)
**Induced sputum**	6 (7.4%)
**BAL fluids and either endotracheal aspiration or induced sputum**	19 (23.5%)

IQR, interquartile range; CMV; cytomegalovirus

†Others: Interstitial lung disease on steroid use, membranous glomerulonephritis, focal segmental glomerulosclerosis

‡ Second-line treatment: primaquine and clindamycin in 18 patients and pentamidine in 1 patient

In 7 patients who had not been intubated, 6 patients presented positive for PCP PCR via sputum sample, and one patient presented positive via BAL fluid. Among 74 patients who received mechanical ventilator treatment, 51 patients presented positive for PCP PCR from a sample of endotracheal aspirates, and 4 patients from BAL fluid. Nineteen patients presented positive for PCP PCR from both BAL fluid and endotracheal aspiration samples.

### Management of PCP

All patients received intravenous or oral trimethoprim (15–20 mg/kg per day) and sulfamethoxazole (75–100 mg/kg per day) as the first-line treatment for PCP. The general and planned treatment period was 3 weeks. All patients also received adjuvant corticosteroid therapy (40 mg prednisone twice daily for 5 days, followed by 20 mg prednisone twice daily every 5 days). Nineteen patients received second-line treatment for not showing clinical improvement, receiving either primaquine (15–30 mg/day) and clindamycin (1800 mg/day) (n = 18) or pentamidine (4 mg/kg per day, n = 1). Among these, only 4 (21.1%) survived; all-survivors had a PCP PCR negative conversion. Six patients (50%) in the group of non-survivors had a PCP PCR-negative conversion. The median time before converting to the second-line regimen was 9.1 ± 2.8 days.

### Outcomes and prognostic factors

The overall survival rate for PCP patients in the ICU was 35.8% (29/81). Seventy-four patients (91.3%) required mechanical ventilation, and 6 patients (7.4%) required high-flow nasal oxygen treatment; among these, there were 24 (32.4%) and 4 (66.7%) survivors, respectively.

On univariate analysis, the following factors significantly correlated with mortality in PCP patients, with the values for survivors and non-survivors, respectively, shown in parentheses: APACHE II score (17.0 and 23.0, *p* < 0.001), renal failure requiring renal replacement therapy (20.7% and 53.8%, *p* = 0.004), PCP PCR-negative conversion (96.6% and 63.5%, *p* = 0.001), and PaO_2_/FiO_2_ ratio in the first 24 hours from MICU admission (275.6 ± 68.3 and 180.7 ± 73.6, *p* < 0.001) (Table 2). White blood cell counts, percent delta neutrophils, and blood urea nitrogen, creatinine, albumin, C-reactive protein, and procalcitonin were not related to mortality in PCP patients.

**Table 2:** Comparison of the characteristics between survivors and non-survivors.

Variable	Survivors (n = 29)	Non-survivors (n = 52)	*p* value
**Median age, years (IQR)**	58.0 (44.0, 70.0)	62.6 (54.0, 72.0)	0.174
**Male sex, n (%)**	17 (58.6%)	34 (65.4%)	0.546
**Combined shock, n (%)**	11 (37.9%)	25 (48.1%)	0.378
**APACHE II score, median (IQR)**	17.0 (13.5, 19.5)	23.0 (18.0, 29.0)	<0.001
**Renal failure requiring RRT, n (%)**	6 (20.7%)	28 (53.8%)	0.004
**Underlying disease, n (%)**			
**Malignancy**	9 (31.0%)	20 (38.5%)	0.504
**Solid organ transplantation**	7 (24.1%)	9 (17.3%)	0.459
**Corticosteroid use history**	18 (62.1%)	27 (51.9%)	0.378
**PCP PCR negative conversion, n (%)**	28 (96.6%)	33 (63.5%)	0.001
**Initial laboratory findings, median (IQR)**			
**White blood cells/μL**	7590 (5830, 11230)	8770 (4740, 15510)	0.389
**% delta neutrophils**	3.40 (1.95, 8.25)	4.90 (2.00, 11.50)	0.259
**mg/mL blood urea nitrogen**	25.8 (15.7, 36.9)	24.2 (12.4, 38.2)	0.969
**mg/mL creatinine**	1.3 (0.6, 2.4)	0.8 (0.5, 1.6)	0.400
**g/mL albumin**	2.7 (2.4, 3.2)	2.5 (2.3, 2.9)	0.150
**mg/L C-reactive protein**	122.8 (79.9, 225.7)	142.3 (98.3, 235.8)	0.953
**ng/mL procalcitonin**	0.61 (0.28, 4.72)	1.66 (0.60, 5.83)	0.127
**PaO_2_/FiO_2_ ratio, first 24 hours**	275.6 ± 68.3	180.7 ± 73.6	<0.001
**CMV quantitative real-time PCR positive in blood sample, n (%)**	12 (41.4%)	31 (59.6%)	0.115

IQR, interquartile range; RRT, renal replacement therapy; CMV, cytomegalovirus.

Gender and variables with a *p*-value < 0.1 in univariate analysis were used to assess the multivariate analysis, which showed that PCP PCR-negative conversion (hazard ratio, 0.433; 95% confidence interval, 0.203–0.928; *p* = 0.031) and the PaO_2_/FiO_2_ ratio in the first 24 hours (hazard ratio, 0.988; 95% confidence interval, 0.983–0.993; *p* < 0.001) independently predicted PCP prognosis (Table 3).

**Table 3:** Factors associated with mortality in non-HIV PCP patients with respiratory failure in a multivariate analysis.

Variable	Hazard ratio	95% CI	*p* value
**Male sex**	1.524	0.810–2.869	0.192
**APACHE II score**	1.001	0.960–1.043	0.976
**Renal failure requiring RRT**	1.371	0.707–2.657	0.351
**PCP PCR negative conversion**	0.433	0.203–0.928	0.031
**PaO₂/FiO₂ ratio, first 24 hours**	0.988	0.983–0.993	<0.001

CI, confidence interval; RRT, renal replacement therapy

### PCP PCR-negative conversion and mortality

**Fig 2:**
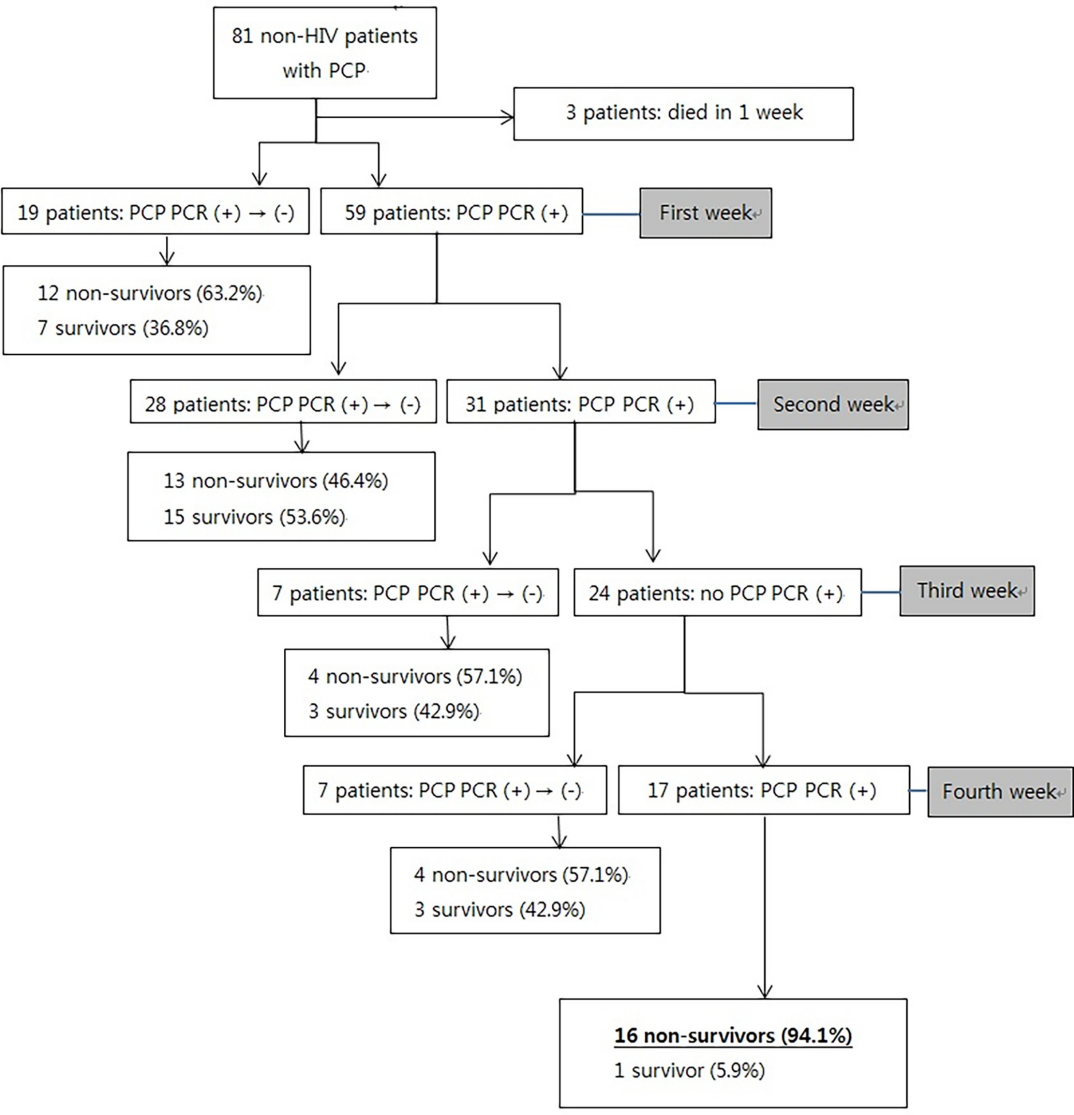
Flowchart showing mortality rates in non-HIV PCP patients with respiratory failure at different times after diagnosis. PCP, jirovecii pneumonia; PCR (+) → (-), polymerase chain reaction negative conversion; BAL, bronchoalveolar lavage.

**Fig 3:**
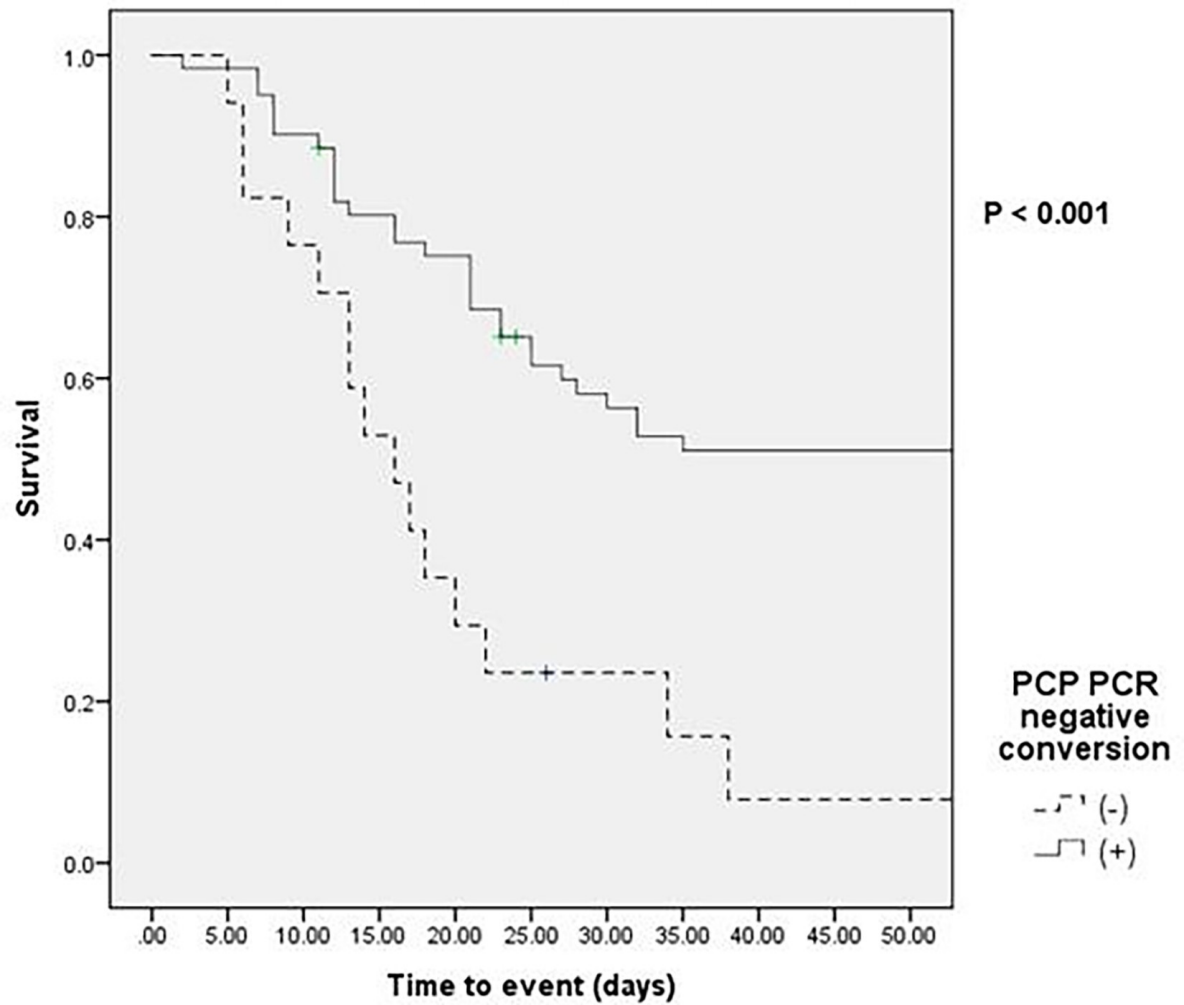
Kaplan-Meier survival curves for non-HIV PCP patients with respiratory failure. Solid line, no PCP PCR conversion. Broken line, PCP PCR negative conversion. PCP, *Pneumocystis jirovecii* pneumonia; PCR, polymerase chain reaction.

Sixty-one patients (78.2%) had a PCP PCR-negative conversion within 4 weeks of diagnosis; 17 patients did not. Three patients died within 5 days and were not tested for following PCP PCR. The median time for conversion was 10.0 (7.0–14.0) days. Negative conversion occurred in 19 patients in the first week after diagnosis, of which 12 (63.2%) did not survive (Fig 2). In the first week, the mortality rate was higher than in following weeks due to the severity of the non-survivors’ illness. The number of patients with renal failure requiring renal replacement therapy was higher in non-survivors (n = 7) than in survivors (n = 0), and the PaO_2_/FiO_2_ ratio in the first 24 hours was lower in non-survivors (180.7 ± 73.6) than in survivors (275.6 ± 73.6). Negative conversion occurred in 28 patients in the second week after diagnosis, of which 13 (46.4%) did not survive. The mortality rate was higher (57.1%) in patients with negative conversion in the third and fourth week after diagnosis (4 and 7 patients, respectively). The mortality rate in the 17 patients without conversion was 94.1%. In a Kaplan-Meier survival analysis, higher PCP PCR-negative conversion rates significantly correlated with lower mortality rates (*p* < 0.001) (Fig 3).

## Discussion

In this study, we identified 2 significant prognostic factors for mortality in non-HIV PCP patients with respiratory failure: the PCP PCR-negative conversion rate and the PaO_2_/FiO_2_ ratio in the first 24 hours. Several previous studies have also identified prognostic factors for PCP in non-HIV patients, but controversy remains [5,13,14].

Diagnosis of PCP is based on the presence of symptoms (cough, fever, and dyspnea) and laboratory and radiological findings [1]. Detecting *P*. *jirovecii* is important, and for this purpose, PCP PCR is more sensitive and specific than conventional staining and routine microscopy [15]. The sensitivity and specificity of PCP PCR for diagnosing PCP in induced sputum, endotracheal aspirates, and BAL fluid in our center were 78% and 93%, respectively. In one study, the sensitivity, specificity, and positive and negative predictive values of PCR for Pneumocystis in BAL fluids were 100%, 91%, 65%, and 100%, respectively [16]. In another, all values were 100% in both BAL fluids and induced sputum [17]. In agreement with other researchers, we believe that PCP PCR is a reliable method for diagnosing PCP in non-HIV high-risk patients. BAL is invasive and costly to perform, while induced sputum and endotracheal aspirates are non-invasive and inexpensively collected.

A good treatment response is usually indicated by the disappearance of symptoms and by hypoxemia (determined by laboratory finding or PaO_2_/FiO_2_ ratio) [9]. Rapid resolution of pulmonary infiltrations on thin-section chest CT also indicates a favorable response; hence, CT is a useful monitoring tool [18]. However, as clinical presentations and the results of imaging or laboratory tests are often inconsistent in determining the treatment response, evaluating the treatment response of PCP pneumonia is difficult.

In a previous study, the overall mortality rate in non-HIV PCP patients was 30.6% with or without respiratory failure [13]. In that study, higher mortality rates and poorer outcomes were associated with older age, female sex, longer time from onset of symptoms to diagnosis, respiratory failure, an underlying solid tumor, high lactate dehydrogenase levels, low serum albumin levels, bacterial or aspergillus infection, and most notably, respiratory failure [13]. In another study, the overall mortality rate in non-HIV PCP patients was 63.3%, and was higher in patients with respiratory failure than in those without [19].

PCP patients without serious illness are managed in general wards or as outpatients. However, acute respiratory failure in these patients worsens their prognosis [19]; the mortality rate in HIV PCP patients with respiratory failure is approximately 60%, which is higher than that in HIV PCP patients without respiratory failure [20]. HIV negativity also worsens prognosis and accelerates disease progression. Therefore, non-HIV PCP patients with acute respiratory failure require intensive management, and detection of treatment failure during early-phase treatment is important.

According to one retrospective review, unsuccessful initial antimicrobial treatment indicates poor prognosis in non-HIV PCP patients with acute respiratory failure [10]. Other reported indicators include a high APACHE III score, delayed intubation, longer duration of invasive positive pressure ventilation, and pneumothorax [21]. Our study correlated low PCP PCR-negative conversion rates and low PaO_2_/FiO_2_ ratios in the first 24 hours with high mortality rates and poor outcomes in non-HIV PCP patients with respiratory failure. Low PaO_2_/FiO_2_ ratios have also been associated with poor outcomes in PCP patients with autoimmune disease [22]. We suggest that the absence of PCP PCR-negative conversion is a valid indicator of treatment failure. When negative conversion does not occur during first-line treatment, second-line treatment should be initiated and the patient should be closely observed.

Lastly, we found that the mortality rate increased as the duration between diagnosis and PCP PCR-negative conversion lengthened. Mortality rates in the second, third, and fourth week after diagnosis in patients with negative conversion were 42.9%, 57.1%, and 57.1%, respectively. The mortality rate in the first week was relatively high (63.2%), presumably because more non-survivors (versus survivors) experienced renal failure requiring renal replacement therapy (*p* = 0.017) during this time. In patients without negative conversion, the overall mortality rate was 94.1%.

Our study has several limitations. First, it was conducted retrospectively at a single center. Secondly, it included patients with severe illness requiring ventilation. This may account for the lower survival rates in our study compared with those in other studies. Thirdly, we only screened for Pneumocystis via PCR; no microbiological staining of respiratory specimens was performed. Hence, some of the patients in our study were considered to the *P*. *jirovecii* colonization. However, according to a previous study, colonization is twice as common in HIV patients than in non-HIV patients, perhaps because of the lower number of the CD4 lymphocytes in the former [23]. As Pneumocystis infection, unlike Pneumocystis colonization, is accompanied by signs and symptoms of pneumonia [24], we only included symptomatic patients in our study. Moreover, the included patients had radiologic findings consistent with PCP infection.

## Conclusions

Our findings identified PCP PCR negative conversion and PaO_2_/FiO_2_ ratio in the first 24 hours as prognostic factors for severe PCP in non-HIV patients with respiratory failure. We suggest that follow-up PCP PCR testing may aid in assessing the effectiveness of initial anti-Pneumocystis treatments. Clinicians in ICUs should closely observe non-HIV PCP patients with low initial PaO_2_/FiO_2_ ratios and repeated PCP PCR positivity. Prospective studies are needed to further examine the role of PCP PCR-negative conversion in non-HIV PCP patients with respiratory failure in order to improve survival rates.

## Supporting information

S1 Dataset (XLSX)
